# SUMOylation in Viral Replication and Antiviral Defense

**DOI:** 10.1002/advs.202104126

**Published:** 2022-01-21

**Authors:** Yao Fan, Xiang Li, Lei Zhang, Zhi Zong, Fangwei Wang, Jun Huang, Linghui Zeng, Chong Zhang, Haiyan Yan, Long Zhang, Fangfang Zhou

**Affiliations:** ^1^ Department of Pharmacology Zhejiang University City College School of Medicine Hangzhou Zhejiang 310015 China; ^2^ Institutes of Biology and Medical Science Soochow University Suzhou 215123 China; ^3^ MOE Laboratory of Biosystems Homeostasis and Protection and Innovation Center for Cell Signaling Network Life Sciences Institute Zhejiang University Hangzhou 310058 China; ^4^ Department of Orthopaedic Surgery The Third Affiliated Hospital of Wenzhou Medical University Rui'an 325200 China

**Keywords:** antiviral immunity, infection, replication, SUMOylation, viral proteins

## Abstract

SUMOylation is a ubiquitination‐like post‐translational modification that plays an essential role in the regulation of protein function. Recent studies have shown that proteins from both RNA and DNA virus families can be modified by SUMO conjugation, which facilitates viral replication. Viruses can manipulate the entire process of SUMOylation through interplay with the SUMO pathway. By contrast, SUMOylation can eliminate viral infection by regulating host antiviral immune components. A deeper understanding of how SUMOylation regulates viral proteins and cellular antiviral components is necessary for the development of effective antiviral therapies. In the present review, the regulatory mechanism of SUMOylation in viral replication and infection and the antiviral immune response, and the consequences of this regulation for viral replication and engagement with antiviral innate immunity are summarized. The potential therapeutic applications of SUMOylation in diseases caused by viruses are also discussed.

## Graphical abstract

This review systematically summarizes the role and mechanisms of SUMOylation in regulating viral replication and antiviral defense. SUMOylation of viral proteins, the effect of viruses on the SUMO pathway, and SUMOylation of antiviral immunity components are concluded. Moreover, the prospects and challenges of utilizing SUMOylation as therapeutic targets for treatment of infectious diseases are discussed.

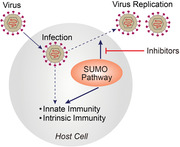

## 1 Introduction

SUMOylation is a dynamic and reversible protein post‐translational modification that is involved in the regulation and diversification of protein function. This modification is achieved by covalently attaching a small ubiquitin‐like modifier (SUMO) to a target protein. Viruses cannot survive and reproduce without hijacking certain cellular pathways from within the living cells of their host. As such, it is unsurprising that viral proteins can exploit the host SUMO system for their benefit. The first reported SUMOylation of viral protein is the human cytomegalovirus (HCMV) immediate‐early 1 (1E1) protein, which was discovered to be SUMOylated in 1999.^[^ ^1^ ^]^ To date, many viral proteins have been shown to be SUMOylated, and the process of SUMOylation is considered to be important for viral replication, as it enhances viral macromolecular synthesis and assembly, and inhibits the host immune response.^[^ ^2^, ^3^, ^4^ ^]^ However, in some cases, the SUMOylation of viral proteins is not conducive to viral replication.^[^ ^5^ ^]^

In addition to the regulation of the SUMOylation of viral proteins, there is emerging evidence that SUMOylation also plays a key role in the ability of host cells to resolve viral infection. The innate and intrinsic immune systems are essential for host to defend against virus invasion, and involve the pre‐existing or inducible antiviral factors (e.g., interferons (IFNs) and inflammatory cytokines) to establish an antiviral environment. Recently, it has been reported that SUMOylation can influence the regulation of type I IFNs (IFN*α* and IFN*β*) in response to viral infection.^[^ ^6^, ^7^, ^8^ ^]^ Viral interference with the host SUMO system can change the global SUMOylation level, which is related to the mechanism by which viruses evade antiviral defense.^[^ ^8^ ^]^ Given the functional diversity of SUMOylation, it is entirely likely that SUMOylation can both enable and inhibit viral infection.

SUMOylation connects the virus and host closely; thus, a deep understanding of the regulatory mechanisms by which SUMOylation affects viral and antiviral defenses could provide insights that enable more effective control of diseases caused by viral infection, and the development of therapeutics based on SUMOylation. In the present review, we briefly review the mechanisms of the SUMO system, and we highlight the regulatory mechanisms of the underlying SUMOylation‐modulated viral replication and host cellular immune system. We critically discuss the possibility that SUMOylation may be useful in the development of antiviral therapeutic strategies. Future studies in this field have the potential to provide new mechanistic insights into host antiviral defenses.

## 2 General Overview of SUMOylation

SUMO proteins were discovered in the 1990s.^[^ ^9^, ^10^ ^]^ Similar to ubiquitination, SUMOylation attaches small polypeptides of ≈12 kDa to the lysine (Lys) residues of target proteins. According to current research, SUMO proteins are highly conserved across all eukaryotes. Five SUMO paralogs have been identified in the human genome (SUMO1–5). SUMO1, 2, and 3 are widely distributed in human tissue, and have been extensively studied,^[^ ^11^ ^]^ while the expression of SUMO4 is limited to the lymph nodes, spleen, and kidneys, and is associated with type 1 and type 2 diabetes.^[^ ^12^, ^13^ ^]^ SUMO5 is restricted to some specific tissues, such as the peripheral blood leukocytes and testes.^[^ ^14^ ^]^ SUMO2 has 97% similarity with SUMO3, but only 47% sequence identity with SUMO1.^[^ ^15^ ^]^ These SUMO isoforms also differ in their ability to form chains, which leads to signal variation in the SUMO pathway.

**Figure 1:**
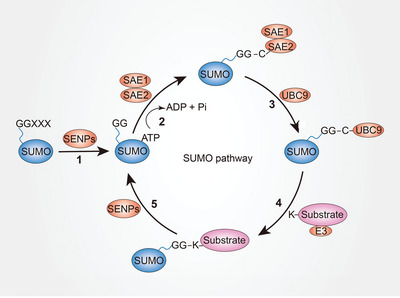
The mechanisms of the SUMO pathway. Step 1: maturation. The small ubiquitin‐like modifier (SUMO) propeptide is cleaved at the C‐terminus by SENPs into a mature form with a Gly–Gly motif. Step 2: activation. SUMO‐activating enzyme (SAE1 and SAE2) interact with the terminal glycine of mature SUMO to form a thioester bond in the presence of ATP. Step 3: conjugation. SUMO protein is transferred to SUMO‐conjugating enzyme UBC9, forming a thioester bond at the C‐terminal glycine of SUMO. Step 4: ligation. UBC9 transfers SUMO protein to the Lys residues of a substrate with the help of SUMO E3 ligase. Step 5: deSUMOylation. The process of SUMOyation can be reversed by SENPs. SENPs can cleave the SUMO terminal glycine from the lysine residues of the substrates.

SUMOylation is carried out through the sequential action of E1‐activating enzymes, E2‐conjugating enzymes, and E3 ligases (**Figure** **1**). Compared to ubiquitin, SUMO has a much more restricted enzymatic toolkit to utilize, and only a single E1‐activating enzyme (heterodimeric SAE1/SAE2) and E2 SUMO‐conjugating enzyme (UBC9) exist. However, several E3 ligases have been discovered (e.g., PIAS, RanBP2, and Pc2).^[^ ^16^, ^17^, ^18^ ^]^ In the process of SUMOylation, SUMO paralogs are expressed as immature propeptides that require cleavage to expose the C‐terminal diglycine motif by SUMO‐specific proteases (SENPs). After cleavage, SUMO can be bound to mature functional proteins to exert its role.^[^ ^19^, ^20^ ^]^ In the subsequent SUMO activation step, the E1 activating enzyme interacts with SUMO to form a thioester bond between the cysteine of SAE2 and the terminal glycine of mature SUMO in the presence of ATP.^[^ ^21^ ^]^ The SUMO protein is then transferred to the cysteine residue of UBC9, forming a thioester bond between the catalytic cysteine of UBC9 and the carboxyl group of the C‐terminal glycine of SUMO.^[^ ^22^, ^23^ ^]^ Finally, with the help of E3 ligase, UBC9 transfers the SUMO protein to the substrate at Lys residues within a specific consensus motif *ψ*KxD/E (where *ψ* represents a hydrophobic residue, and x is any amino acid).^[^ ^24^ ^]^ However, not all SUMO substrates have this motif.^[^ ^25^ ^]^ Similar to ubiquitination, the process of SUMOylation is also reversible. Various SUMO‐specific proteases are responsible for removing the SUMO terminal glycine from the lysine residues of the substrates. Currently, there are three known classes of SUMO proteases: the ubiquitin‐like protease/sentrin‐specific protease (Ulp/SENP) family, the deSUMOylating isopeptidase (Desi) family, and ubiquitin‐specific peptidase‐like protein 1 (USPL1).^[^ ^26^ ^]^ Ulp1 and Ulp2 are two proteins that function in yeast and prokaryotes, while SENPs function in mammals.^[^ ^27^ ^]^ There are six SENPs that function as SUMO proteases, including SENP1‐3 and SENP5‐7. DeSI1, DeSI2, and USPL1 are also SUMO proteases that have minimal sequence similarity, different structures, and specific substrates with SENPs.^[^ ^28^, ^29^ ^]^ Each SUMO protease has a catalytic domain, which is responsible for its protease function, with key histidine and cysteine residues. Substrates with characteristic SUMO chains are recognized by incorporated factors, including SUMO‐interacting motifs (SIM), and activate downstream signaling.^[^ ^30^ ^]^

## 3 Regulation of Viral Replication and Infection by SUMOylation

After infecting the host cell, viruses need to hijack components of the host machinery to replicate. There are multiple mechanisms by which viruses manipulate the process of SUMOylation. These can be broadly categorized into viral proteins that are SUMOylated, and/or viruses that affect the SUMO pathway.^[^ ^31^ ^]^

### 3.1 SUMOylation of Viral Proteins

HCMV is a member of the herpesvirus family that can cause life threatening to the immunocompromised individuals. HCMV IE1 protein is the first protein expressed during HCMV infection, which is involved in disrupting host antiviral responses and regulating expression of viral genes. IE1 has been previously shown to be SUMOylated by SUMO 1 at Lys 450.^[^ ^32^, ^33^ ^]^ The SUMOylation‐deficient mutant virus K450R displayed attenuated growth and generated a lower viral titer.^[^ ^32^ ^]^ Although the lack of SUMOylation did not affect the subcellular localization of IE1 and its ability to disrupting promyelocytic leukemia protein (PML) nuclear bodies (NBs), SUMOylation‐deficient IE1 reduced expression levels of the IE2 and resulted in an impaired capacity for HCMV replication.^[^ ^32^ ^]^ Conversely, the SUMOylation of IE1 at Lys450 has also been reported to suppress the interaction between IE1 and STAT2, which relieves the repressive effect of IE1 on IFN‐regulated gene expression.^[^ ^34^ ^]^

Zika virus (ZIKV) and dengue virus (DENV) are two mosquito‐borne flaviviruses. The NS5 protein of flaviviruses is a viral methyltransferase and RNA‐dependent RNA polymerase, which is essential for RNA capping, replication of the viral genome, and suppression of the host interferon response.^[^ ^35^ ^]^ Recently, it has been reported that flavivirus‐specific SIM in NS5 proteins directs its nuclear localization, and the SUMOylation of ZIKA NS5 protein determines its assembly into discrete NBs.^[^ ^36^ ^]^ During ZIKV infection, the SUMOylation of NS5 at Lys 252 promotes NS5 NB interactions with STAT2, thereby further disrupting the antiviral PML‐STAT2 NBs, promoting PML degradation, and inhibiting the interferon‐stimulated gene (ISG) response, which finally results in the persistent infection of human brain microvascular endothelial cells.^[^ ^36^ ^]^

Matrix 1 (M1) protein of influenza A virus (IAV) provides support underneath the envelope proteins. The SUMOylation of M1 is a typical example of a SUMOylated viral protein that facilitates viral assembly. M1 plays a critical role in IAV assembly by interacting with viral ribonucleoproteins (vRNPs), which are complexes containing viral RNA from eight gene segments.^[^ ^37^ ^]^ The SUMOylation of M1 at Lys 242 is necessary for efficient interactions between M1 and vRNPs to form the M1‐vRNPs complex.^[^ ^38^ ^]^ The lack of M1 SUMOylation leads to the accumulation of viral RNA and viral proteins in cells, and prevents subsequent viral morphogenesis. Viruses carrying SUMO‐deficient M1 produce a lower viral titer.^[^ ^38^ ^]^

Human papillomaviruses (HPV) are DNA tumor viruses that cause cervical, anal, and oropharyngeal carcinomas by infecting cutaneous and mucosal epithelia. The HPV E2 protein is required for controlling viral replication, transcription, and viral genome segregation.^[^ ^39^ ^]^ The HPV‐16 E2 protein can be SUMOylated at Lys 292.^[^ ^40^ ^]^ The mutation of Lys 292 to Arg results in the loss of its ability to be SUMOylated, and prevents transcription, although the DNA binding activity is unchanged.^[^ ^40^ ^]^ The SUMOylation of E2 protein may also contribute to increased replication by enhancing its stability.^[^ ^41^ ^]^ The E1 protein is another viral protein that is required for replication of the viral genome. Bovine papillomavirus (BPV) E1 is SUMOylated by covalent attachment of SUMO‐1 at Lys 514.^[^ ^42^ ^]^ Loss of SUMOylation of the BPV E1 protein prevents its normal nuclear accumulation, resulting in an impaired capacity for replication.^[^ ^42^ ^]^

The above evidences indicate that SUMOylation plays a significant role in regulating viral replication and infection via a wide variety of mechanisms, such as inhibition of host antiviral immunity, regulation of protein interaction, localization, stability, and transcriptional activity. SUMOylation has been identified in numerous viral proteins (**Table** **1**), and there appears to be no single role for SUMOylation in modulating viral protein function. Thus, an in‐depth understanding of how SUMOylation regulates the function of viral proteins is critical for developing methods to treat diseases caused by viruses.

**Table 1:** Examples of SUMOylated viral proteins

Virus family	Virus	Protein	SUMO site	Function of SUMOylation	Refs.
Flaviviruses	ZIKA	NS5	K252	Disrupt the formation of PML‐STAT2 NBs and inhibit the induction of ISGs	^[^ ^36^ ^]^
	DENV	NS5	K546	Regulate its nuclear localization	^[^ ^36^ ^]^
Orthomyxovirus	Influenza A viruses	M1	K242	Required for forming the M1‐vRNPs complex	^[^ ^38^ ^]^
		NS1	K131 K221	Required for the rapid replication of H1N1 influenza viruses; Enhance its protein stability and accelerate virus replication	^[^ ^43^, ^44^ ^]^
		NP	K4/K7	Required for its intracellular trafficking and for virus growth	^[^ ^45^ ^]^
		PB1	K612	Keep its ability to bind viral RNA	^[^ ^46^ ^]^
Coronavirus	SARS‐CoV‐1	NP	K62	Promote its homo‐oligomerization and disrupt the division of host cell	^[^ ^47^ ^]^
Retrovirus	HIV	integrase	Unclear	Inhibit the viral genome integration of HIV	^[^ ^48^ ^]^
		p6	K27	Reduce the infectivity of the released HIV‐1 virions	^[^ ^49^ ^]^
	HTLV	Tax	Multiple	Determine its nuclear localization and activation of NF‐kB pathway	^[^ ^50^, ^51^, ^52^ ^]^
		APH‐2 (HTLV‐1)	Unclear	Required for PML‐NBs nuclear localization and control its stability	^[^ ^53^ ^]^
Filovirus	Ebola	VP40	K326	Regulate its stability	^[^ ^54^ ^]^
		VP24	K14	Regulate its stability	^[^ ^55^ ^]^
Papillomavirus	BPV	E1	K514	Promote nuclear accumulation of E1	^[^ ^42^ ^]^
	HPV	E1	Unclear	Unclear	^[^ ^42^ ^]^
		E2	K292	Enhance its stability	^[^ ^40^ ^]^
		L2	K35	Inhibit its binding to capsid protein L1	^[^ ^56^ ^]^
Herpesvirus	EBV	BZLF1	K12	Suppress its transcriptional activity	^[^ ^57^ ^]^
		BRLF1/Rta	K19/K213/K517	Enhance its transactivation activity	^[^ ^58^ ^]^
		EBNA3C	Unclear	Required for its coactivation activity with EBNA2	^[^ ^59^ ^]^
Herpesvirus	KSHV	K‐bZIP	K158	Required for its transcriptional repression activity	^[^ ^60^ ^]^
		LANA1 (Orf36)	K1140	SIMs are required for recruitment cellular proteins	^[^ ^61^ ^]^
		LANA2 (vIRF3)	K57	Disrupt the formation of PML‐NBs	^[^ ^62^ ^]^
	CMV	IE1	K450	Suppress its binding to STAT2	^[^ ^33^, ^34^ ^]^
		IE2p86	K175/K180	Enhance its transactivation activity	^[^ ^63^ ^]^
		UL44	K410	Enhance virus production and DNA replication	^[^ ^64^, ^65^ ^]^
Adenovirus	AdV	E1B 55K	K104	Regulate the NES‐dependent nuclear export of the AdV protein	^[^ ^66^, ^67^ ^]^
Poxvirus	Vaccinia	E3	K40/K99	Suppress its transcriptional activity	^[^ ^68^ ^]^

### 3.2 The Effect of Viruses on the SUMO Pathway

During infection and replication, viruses can manipulate the entire process of SUMOylation, resulting in global changes in cellular SUMOylation levels. SUMOylation is initiated by the expression of SUMO genes and the maturation of the SUMO propeptide. Epstein‐Barr virus (EBV) latent membrane protein‐1 (LMP1) can induce the expression of SUMO1/2/3 in EBV‐positive cell lines, and this induction requires the activation of nuclear factor‐*κ*B (NF‐*κ*B) signaling through C‐terminal activating regions1 (CTAR1) and 2 (CTAR2) of LMP1.^[^ ^69^ ^]^ Hepatitis C virus (HCV) infection can also increase the expression of SUMO1, which is responsible for HCV replication.^[^ ^70^ ^]^ These studies indicate that viruses can regulate the SUMOylation process by controlling SUMO expression levels.

After SUMO paralogs are translated and cleaved to maturation, SUMO proteins bind with SAE1/2 to form the E1‐SUMO intermediate, while viruses can impair this formation by inactivating SAE1/2 in some cases. For example, the adenoviral protein Gam1 can recruit Cul2/5‐EloB/C‐Roc1 ubiquitin ligase complexes to SAE1/2, which leads to SAE1 ubiquitylation and degradation by the proteasome.^[^ ^71^ ^]^ With the degradation of SAE1, SAE2 becomes unstable and is then degraded by the proteasome.^[^ ^71^ ^]^ Thus, the activity of SAE1/2 is also a target for viruses to manipulate the process of SUMOylation.

Given that UBC9 functions as the only E2 SUMO‐conjugating enzyme in the SUMO pathway, it is an ideal target for viruses to modulate the host SUMO system. For example, LMP1 is a latency‐associated protein of EBV that plays a key role in maintaining viral latency. LMP1 can induce the SUMOylation of cellular proteins by interacting with UBC9 via CTAR3.^[^ ^72^ ^]^ KRAB‐associated protein‐1 (KAP1) is a target of LMP1‐induced SUMOylation, and is required for maintaining the latency of EVB through transcriptional repression. Inhibition of LMP1‐induced SUMOylation abrogates KAP1 function and disrupts the latency of EBV.^[^ ^72^ ^]^ HPV E6 proteins are another example of an interaction with UBC9.^[^ ^73^ ^]^ E6 can bind to UBC9 and mediate UCB9 proteasome degradation, and the resulting reduction in UCB9 causes decreased host SUMOylation, and accelerates the development of cervical cancer.^[^ ^73^ ^]^ There are multiple mechanisms by which viruses utilize host E2 SUMO‐conjugating enzymes. Viruses can induce or inhibit the global SUMOylation of cellular proteins by targeting UBC9.^[^ ^73^, ^74^ ^]^ they can be SUMOylated by interacting with UBC9.^[^ ^40^, ^75^ ^]^ and they can hijack UBC9 independent of the SUMOylation process.^[^ ^76^, ^77^ ^]^ A better understanding of these mechanisms could be beneficial for developing more effective treatment of viral diseases.

Similar to ubiquitination, SUMO E3 ligases catalyze the transfer of SUMO from UBC9 to a substrate with substrate specificity. For some viruses, the process of SUMOylation can be catalyzed by their own encoded E3 ligase. The adenoviral early protein region 4 (E4)‐ORF3 functions as a SUMO E3 ligase of transcriptional intermediary factor‐1 gamma (TIF‐1*γ*), which is involved in transcriptional regulation, TGF‐*β* signaling, and DNA damage repair.^[^ ^78^, ^79^ ^]^ E4‐ORF3 induces the SUMOylation of TIF‐1*γ* with specificity toward SUMO3, and promotes poly‐SUMO chain elongation, finally leading to its degradation by the proteasome.^[^ ^78^ ^]^ A recent study published by the same group showed that E4‐ORF3 SUMO ligase activity is conserved across human adenovirus lineages, and E4‐ORF3‐mediated SUMOylation of target proteins occurs only when they are recruited into E4‐ORF3 nuclear inclusions in infected cells.^[^ ^80^ ^]^ In addition, in vitro SUMO conjugation assays revealed that the adenoviral protein E1B‐55 K is also a target of E4‐ORF3‐mediated SUMOylation.^[^ ^80^ ^]^ However, viruses also manipulate the SUMOylation process by affecting SUMO E3 ligases in the host cells. For example, the adenoviral E4‐ORF3 specifically targets and mislocalizes the protein inhibitor of activated STAT3 (PIAS3) to facilitate infection, suggesting that the SUMOylation processes can be redirected by viral infection to promote viral replication and infection.^[^ ^81^ ^]^ The mechanisms by which viruses interact with SUMO E3 ligases are complex and diverse. Viruses can alter the location of SUMO E3 ligases,^[^ ^81^ ^]^ increase or decrease the expression level of SUMO E3 ligases,^[^ ^82^ ^]^ or interact with SUMO E3 ligase‐independent SUMOylation processes.^[^ ^83^, ^84^ ^]^ To date, the regulatory mechanism of E3 by viruses remains unclear, and requires further investigation.

The final step of the SUMOylation process is the removal of the SUMO proteins from the substrate by SENPs, which are also responsible for mediating the maturation of SUMO proteins. Viruses can disrupt these two processes via SENPs, thereby redirecting SUMOylation. A recent study suggested that LMP1 inhibits the ubiquitination of SENP2, which results in stabilizing SENP2 expression, suppressing SENP2 activity, and reducing the nuclear localization of SENP2.^[^ ^85^ ^]^ Finally, the functional consequence of SENP2 ubiquitination is the decreased deSUMOylation of cellular proteins, which contributes to the global increase in SUMOylated proteins during latent EBV infections.^[^ ^85^ ^]^ Viral infection can also result in the suppression of viral genome replication by modulating the host SUMOylation machinery. Hepatitis B virus (HBV) infection is the main cause of hepatitis B and hepatocellular carcinoma (HCC). HBV infection reduces SENP3 expression levels. Downregulation of SENP3 increases SUMOylation and degradation of the IQ motif containing GTPase activating protein 2 (IQGAP2), a Ras GTPase‐activating‐like protein, which has been identified as a substrate for SENP3‐mediated deSUMOylation.^[^ ^86^ ^]^ Degradation of IQGAP2 relieves its inhibition of Akt phosphorylation, which suppresses HBV genome replication and restores host protein translation.^[^ ^86^ ^]^ Studies on the mechanisms by which viruses affect SENPs have offered valuable insights toward the development of antiviral treatments targeting SENPs.

**Figure 2:**
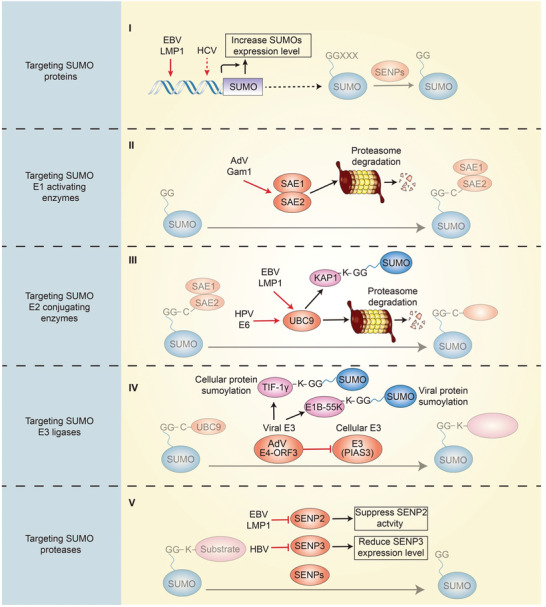
The effect of viruses on the SUMO pathway. (I) Viruses can change the expression level of SUMO proteins. (II) Viral proteins can mediate the degradation of SAE1/2. (III) Viral proteins can induce the SUMOylation of cellular proteins by targeting UBC9 and mediating its degradation. (IV) Viral proteins can act as SUMO E3 ligases to catalyze the SUMOylation of host or viral proteins and inhibit cellular SUMO E3 ligase. (V) Viral infection can inhibit the expression level and activity of SENPs.

Viruses rely heavily on host SUMOylation machinery throughout the viral replication cycle. Through the induction and inhibition of global changes in SUMOylation levels,^[^ ^87^ ^]^ viruses interact extensively with the SUMO system to facilitate their successful infection and replication (**Figure** **2**). These interactions generally support viral infection, although there are some that contribute to modulating the suppression of viral replication and infection, and stimulate of the antiviral responses. Besides, viruses can also evade the antiviral response by targeting the SUMOylated proteins. Typical case in this area is the viral ubiquitin ligase ICP0 of HSV‐1, which has the SUMO Targeting Ubiquitin Ligase (STUbL) properties to induce proteasome‐dependent degradation of SUMOylated proteins.^[^ ^88^ ^]^ PML‐NBs, which play a critical role in intrinsic immunity, are involved in ICP0 targeting, thereby the intrinsic antiviral resistance to HSV‐1 infection is counteracted.^[^ ^88^ ^]^ Exploring new therapeutic strategies that target SUMOylation processes to suppress the replication and pathogenesis of viruses requires further clarification of the mechanisms by which viruses manipulate the SUMOylation machinery.

## 4 SUMOylation Is a Vital Regulator of Antiviral Innate and Intrinsic Immunity

**Figure 3:**
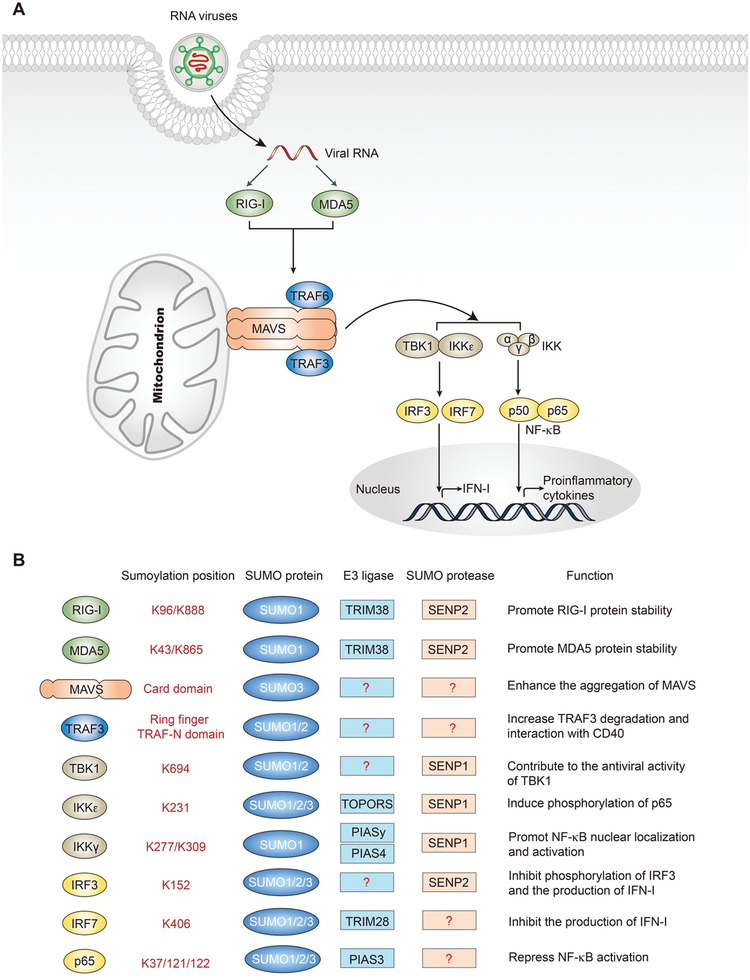
SUMOylation of the RLR signaling pathway. A) The RLR signaling pathway. RIG‐I and MDA5 sense viral RNA and bind to the adapter protein MAVS, leading to MAVS aggregation and recruitment of TRAFs. The IKK complex and TBK1/IKK*ε* are then activated to phosphorylate the transcription factors NF‐*κ*B and IRF3/IRF7, which then translocate to the nucleus to induce the transcription of proinflammatory cytokines and type I interferons. B) SUMOylation of the components of the RLRs signaling pathway.

**Figure 4:**
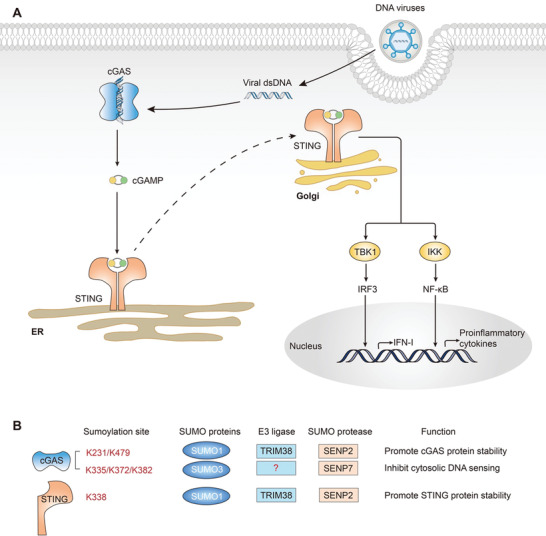
SUMOylation of the cGAS–STING pathway. A) The cGAS‐STING signaling pathway. cGAS senses dsDNA and synthesizes cGAMP as the secondary messenger to transduce the signal to the adapter protein STING. STING is activated and translocates from the ER to the Golgi apparatus, leading to the recruitment and phosphorylation of the transcription factors IRF3 and NF‐*κ*B through the TBK1 and IKK complex, which ultimately triggers the production of type I interferons and proinflammatory cytokines. B) SUMOylation of cGAS and STING.

The innate and intrinsic immune systems play a crucial role in host defense against viral infections. Within the cytosol of mammalian cells, viral nucleic acid is sensed by a limited number of pattern‐recognition receptors, the activation of which triggers diverse signaling pathways.^[^ ^89^ ^]^ The RIG‐I‐like receptor (RLR) pathway, for the recognition of RNA, and the cGAS–stimulator of interferon genes (STING) pathway, for the recognition of DNA, are two important signaling pathways that cause the transcriptional factors IRF3 and NF‐*κ*B to activate and translocate into the nucleus to induce antiviral effector proteins, including type I interferons (IFNs) and other cytokines, which are critical mediators of antiviral innate immune and inflammatory responses.^[^ ^90^, ^91^ ^]^ SUMOylation of antiviral innate immune components affects their activity and function, for example, by altering protein stability and subcellular localization.^[^ ^92^ ^]^ Numerous studies have demonstrated the significance of SUMOylation in antiviral signaling. The regulatory role of SUMOylation on antiviral innate immunity can be broadly categorized based on the viral nucleic acid: the role of SUMOylation in antiviral defense against RNA virus (**Figure** **3**) and the role of SUMOylation in antiviral defense against DNA virus (**Figure** **4**).

Different from innate immune response, intrinsic antiviral resistance is conferred by intrinsic antiviral factors, which can not only detect viral infections but also exert antiviral activities directly.^[^ ^93^, ^94^ ^]^ They usually inhibit viral replication directly without activating the antiviral signal cascade. For instance, IFIT family proteins can recognize viral RNA that contains a 5′‐triphosphate or lacks 2′‐*O*‐methylation, to inhibit viral translation and replication.^[^ ^95^, ^96^, ^97^ ^]^ APOBEC3G plays the function of genome editing during HIV reverse transcription, leading to the reduction of virus replication.^[^ ^98^, ^99^ ^]^

### 4.1 The Role of SUMOylation in the Innate Immune Response to RNA Viruses

The sensing of foreign RNA is mainly mediated by RLRs, including RIG‐I, melanoma differentiation‐associated protein 5 (MDA5), and laboratory of genetics and physiology 2 (LGP2).^[^ ^100^ ^]^ RIG‐I is SUMOylated by SUMO1, and this modification enhances IFN‐I production by increasing its Lys 63 ubiquitylation, and interacting with its downstream adaptor molecule mitochondrial antiviral‐signaling protein (MAVS).^[^ ^101^ ^]^ A recent study found that TRIM38 acts as a SUMO E3 ligase to SUMOylate RIG‐I and MDA5.^[^ ^102^ ^]^ The SUMOylation of RIG‐I and MDA5 suppressed K48‐linked polyubiquitination and degradation in uninfected cells or early infected cells.^[^ ^102^ ^]^ During the late phase of viral infection, SENP2 deSUMOylates RIG‐I and MDA5, resulting in their proteasomal degradation, thus ensuring timely termination of the antiviral response.^[^ ^102^ ^]^ LGP2 has the opposite effects on RIG‐I and MDA5,^[^ ^103^ ^]^ and no study to‐date has demonstrated the SUMOylation of LGP2. Both RIG‐I and MDA5 activate antiviral signaling pathways through MAVS.^[^ ^104^, ^105^, ^106^, ^107^ ^]^ Poly(dA:dT), a repetitive synthetic double‐stranded DNA sequence of poly (dA‐dT):poly (dT‐dA) and a synthetic analog of B‐DNA, induces the formation of SUMO3‐conjugated chains at the card domain of MAVS, which enhances the aggregation of MAVS and drives the secretion of IFN‐*β* in human keratinocytes.^[^ ^108^ ^]^ After activation by RLRs, MAVS recruits and binds to TRAF proteins to promote the activation of the TANK‐binding kinase 1 (TBK1) complex (containing TBK1, I*κ*B kinase‐*ε* (IKKɛ), and IKK *γ* (also known as NEMO)) and the IKK complex (containing IKK*α*/*β* and *γ* ).^[^ ^105^, ^109^ ^]^ The TBK1 complex induces phosphorylation and homodimerization of IRF3 and/or IRF7, which then translocate to the nucleus to promote the expression of type I IFNs.^[^ ^110^, ^111^, ^112^ ^]^ The IKK complex activates NF‐*κ*B to promote the transcription of proinflammatory cytokines.^[^ ^113^ ^]^ The SUMOylation of TRAF3 increases its own protein degradation and interaction with CD40, which leads to noncanonical NF‐*κ*B activation.^[^ ^114^ ^]^ In addition, SUMOylation at Lys 694 of TBK1 enhances its antiviral activity. SENP1 can remove the SUMOylation at this site, and the adenoviral protein Gam1 antagonizes this posttranslational modification.^[^ ^115^ ^]^ IKKɛ can also be SUMOylated by TOPORS at Lys 231, leading to phosphorylation of p65, which contributes to the antiapoptotic function of NF‐*κ*B in response to DNA damage.^[^ ^116^ ^]^ For the IKK complex, SUMOylation of IKK*γ* can be mediated by both the protein inhibitor of activated STATy (PIASy)^[^ ^117^ ^]^ and the protein inhibitor of activated STAT4 (PIAS4),^[^ ^118^ ^]^ while the functions of these two E3‐mediated SUMOylation are distinct. PIASy mediates SUMOylation of IKK*γ* at Lys 277 and 309 by SUMO1, which is required for NF‐*κ*B activation in response to genotoxic agents.^[^ ^117^ ^]^ However, PIAS4 promotes tumorigenicity and metastasis of HCC cells by promoting the SUMOylation of AMPK*α* and IKK*γ*.^[^ ^118^ ^]^ SENP1 mediates IKK*γ* deSUMOylation at Lys 277/309, and the loss of SENP1 leads to increased NF‐*κ*B activity and cytokine production, and induces the inflammatory response.^[^ ^119^, ^120^ ^]^ Vesicular stomatitis virus (VSV) infection induces the SUMOylation of IRF3 and IRF7 conjugated by SUMO1, SUMO2, and SUMO3.^[^ ^8^ ^]^ The mutant K152R of IRF3 and the mutant K406R of IRF7 have lost their SUMO modifications, and result in higher levels of IFN mRNA induction after viral infection.^[^ ^8^ ^]^ P65 (also known as RelA) is SUMOylated by PIAS3, which is induced by NF‐*κ*B activation.^[^ ^121^ ^]^ However, PIAS3‐mediated RelA SUMOylation represses NF‐*κ*B transcriptional activation, as a negative feedback mechanism.^[^ ^121^ ^]^

Toll‐like receptors (TLRs) are also involved in viral RNA sensing in endosomes. There are ten human TLRs, among which TLR3 and TLR7/8 are responsible for RNA sensing. The downstream adapter proteins TRIF and MyD88 are activated by TLRs, and then induce the activation of NF‐*κ*B signaling.^[^ ^122^, ^123^ ^]^ SUMOylation regulates TLR signaling mainly through the modification of components of NF‐*κ*B signaling. SENP6 can remove SUMO2/3 from IKK*γ* to negatively regulate TLR signaling.^[^ ^124^ ^]^ SUMOylation also plays an important role downstream of IFN antiviral defense, which has recently been the focus of a large amount of work.^[^ ^125^, ^126^ ^]^

### 4.2 The Role of SUMOylation in the Innate Immune Response to DNA Viruses

Cyclic GMP–AMP synthase (cGAS), which belongs to the nucleotidyltransferase family, is a mammalian cytosolic DNA sensor that detects pathogenic DNA in the cytoplasm.^[^ ^127^ ^]^ SUMOylation of murine cGas by ubiquitin ligase Trim38 at Lys217 and Lys464, which correspond to Lys231 and Lys479 in human cGAS, occurs in uninfected cells and during the early phase of viral infection.^[^ ^128^ ^]^ The SUMOylation of murine cGas promotes its stability by inhibiting k48‐linked polyubiquitination and degradation.^[^ ^128^ ^]^ After binding to foreign DNA, cGAS initiates signal transduction via the synthesis of the secondary messenger molecule cGAMP.^[^ ^129^, ^130^ ^]^ cGAMP binds to the endoplasmic reticulum (ER) membrane protein “STING”, which is the downstream effector of cGAMP in mammalian cells, which transforms the activity of cGAS into distinct cellular effector responses.^[^ ^127^, ^131^ ^]^ Trim38 also SUMOylates murine Sting at Lys337, which corresponds to Lys338 in human STING, during the early phases of viral infection, promoting both murine Sting protein stability and activation.^[^ ^128^ ^]^ In the late phases of infection, murine Senp2 deSUMOylates cGas and Sting, subsequently leading to proteasomal degradation and the initiation of chaperone‐mediated autophagy pathways.^[^ ^128^ ^]^ However, SUMOylation of cGAS at different sites, Lys335, 372, and 382, can inhibit its DNA‐binding, oligomerization, and nucleotidyltransferase activities.^[^ ^132^ ^]^ SENP7 can abrogate this inhibition by catalyzing cGAS deSUMOylation.^[^ ^132^ ^]^ Upon cGAMP binding, STING forms a multimer and translocates from the ER to the ER‐Golgi intermediate compartment or Golgi apparatus.^[^ ^133^, ^134^, ^135^ ^]^ At the Golgi apparatus, activated STING leads to the recruitment and phosphorylation of the transcription factors IRF3 and NF‐*κ*B through the TBK1 and IKK complex, which ultimately triggers the production of type I interferons and proinflammatory cytokines.^[^ ^127^, ^136^, ^137^, ^138^ ^]^ In addition to the cGAS–STING pathway, IFN‐*γ*‐inducible protein 16 (IFI16),^[^ ^139^ ^]^ DEAD‐box helicase 41 (DDX41),^[^ ^140^ ^]^ DNA‐dependent protein kinase (DNA‐PK),^[^ ^141^ ^]^ meiotic recombination 11 homolog A (Mre11),^[^ ^142^ ^]^ and TLR9^[^ ^143^ ^]^ are DNA sensors that can trigger the innate immune response against DNA viruses. The role of SUMOylation in these signaling molecules remains unclear, and needs further investigation.

### 4.3 The Role of SUMOylation in the Intrinsic Antiviral Resistance

Some excellent examples of SUMOylation regulating intrinsic antiviral immunity come from PML‐NBs. PML‐NBs are composed of a PML protein shell and various partner proteins inside.^[^ ^144^ ^]^ PML, also known as TRIM19, belongs to TRIM family, which is characterized by an RBCC motif composed of a RING domain, a B‐box and a coiled‐coil domain.^[^ ^145^ ^]^ When various herpesviruses such as HSV‐1 or HCMV enter the nucleus, PML‐NBs can induce epigenetic silencing of the viral genome, thereby inhibiting viral replication.^[^ ^146^, ^147^, ^148^ ^]^ Evidences have extended the restricting activity of PML‐NBs to multiple DNA viruses, including adenoviruses, papillomaviruses, and parvoviruses.^[^ ^149^, ^150^, ^151^ ^]^ RNA viruses like LCMV, VSV or HIV‐1 are also involved in the restriction by PML‐NBs.^[^ ^147^, ^152^, ^153^ ^]^ Besides, PML‐NBs also play as coactivator of ISGs and cytokines.^[^ ^154^, ^155^, ^156^ ^]^ It has been reported that SUMOylation is crucial for the assembly of PML‐NBs. PML is SUMOylated at K65, K160, and K490, and carries a SIM.^[^ ^157^, ^158^ ^]^ Numerous PML partner proteins are also modified by SUMOs. SUMO pathway proteins are widespread in PML‐NBs. In fact, apart from SUMO4, all SUMO paralogs are identified in PML‐NBs. UBC9, SENPs, and several SUMO E3 ligases, such as RANBP2 and PIASy, are found to be colocated with PML.^[^ ^159^, ^160^ ^]^ PML is also observed to have SUMO E3 ligase activity.^[^ ^161^, ^162^ ^]^ Generally, SUMOylation hardly affects the formation of the PML shell. However, deficiency of PML SUMOylation abrogates recruitment of partner proteins like DAXX and SP100.^[^ ^163^ ^]^ Moreover, SUMOylation is also required in the accumulation of PML‐NBs on the sites associated with viral genome after viruses enter the nucleus.^[^ ^164^, ^165^ ^]^ Nevertheless, the SUMO‐enriched feature of PML‐NBs is also a natural target for viruses to escape from the intrinsic antiviral resistance. As mentioned above, ICP0 of HSV‐1 can induce proteasome‐dependent degradation of SUMOylated PML‐NBs through its SUMO Targeting Ubiquitin Ligase (STUbL) properties.^[^ ^88^ ^]^ Additionally, IE1 of HCMV interacts with the coiled‐coil domain of PML and disrupts PML‐NBs by abrogating the de novo SUMOylation of PML.^[^ ^166^ ^]^

## 5 SUMOylation: A Potential Target for Antiviral Therapies

**Figure 5:**
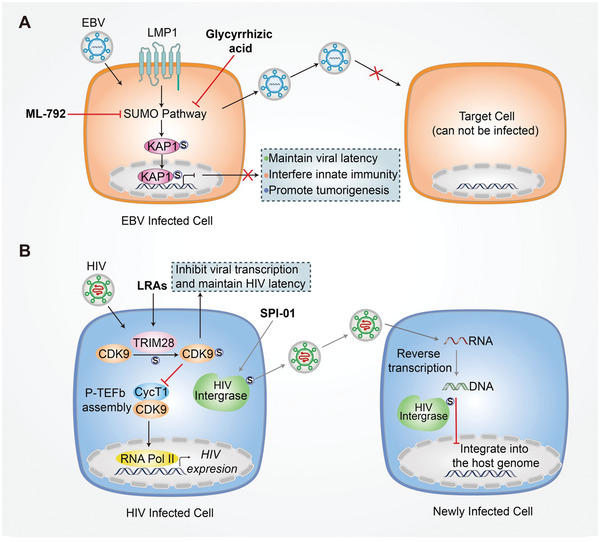
Targeting SUMOylation for antiviral therapy. A) In EBV positive cells, LMP1 upregulates the cellular SUMO pathway to maintain viral latency, interfere with the innate immune response, and promote tumorigenesis. By preventing this, for example, with glycyrrhizic acid or ML‐792, the SUMOylation pathway can be inhibited to suppress the infection of new cells and EBV‐associated lymphoid malignancies. B) In HIV infected cells, TRIM28 SUMOylates CDK9 to inhibit viral transcription and maintain HIV latency through the suppression of P‐TEFb assembly by suppressing interactions between CDK9 and CycT1, which can be targeted by LRAs. The SENP inhibitor SPI‐01 could target HIV integrase to inhibit HIV from integrating into the host genome without influencing its reverse transcription.

Given that SUMOylation has emerged as a key post‐translational modification that can be used by viruses or hosts to alter viral replication and the antiviral response, it could be an ideal drug target for antiviral therapies. Some attempts have been made to target SUMOylation in antiviral therapies, which are summarized in **Figure** **5**.

Latent EBV infection is associated with different lymphoid malignancies, including post‐transplant lymphoproliferative disease and AIDS‐related central nervous system lymphoma.^[^ ^167^ ^]^ LMP1 of EBV dysregulates cellular SUMOylation processes to maintain viral latency, interfere with the innate immune response, and promote tumorigenesis.^[^ ^69^, ^72^ ^]^ Glycyrrhizic acid, a triterpene from licorice root, suppresses the infection of new cells by the virus produced in LMP1‐expressing, EBV‐transformed lymphoblastoid cell lines by inhibiting cellular SUMOylation processes.^[^ ^168^ ^]^ Moreover, a specific and selective small‐molecule inhibitor of SUMOylation (ML‐792) was identified recently, which could inhibit EBV‐associated lymphoid malignancies by suppressing SUMOylation processes in multiple EBV‐positive B cell lines and EBV‐positive nasopharyngeal carcinoma cells.^[^ ^169^, ^170^ ^]^ These results indicate that inhibiting cellular SUMOylation can facilitate the treatment of EBV‐associated lymphoproliferative diseases.

Recent research suggests that TRIM28 (also known as KAP1) acts as a SUMO E3 ligase to SUMOylate cyclin‐dependent kinase 9 (CDK9) with SUMO4 at Lys 44, Lys56, and 68 residues, which prevents P‐TEFb assembly by directly blocking the interaction between CDK9 and Cyclin T1 (CycT1), and consequently inhibits viral transcription, contributing to HIV‐1 latency.^[^ ^171^ ^]^ Hence, targeting TRIM28 and its SUMOylation pathway could provide a new direction for developing efficient latency‐reversing agents (LRAs) for treating HIV infection. Interestingly, a chemotype of the SENP inhibitor, SPI‐01, can inhibit HIV replication.^[^ ^172^ ^]^ The treatment of cells with SPI‐01 results in the production of viral particles that can enter cells and undergo reverse transcription, but cannot effectively integrate into the host genome, which may also provide insights for the development of strategies to cure HIV.^[^ ^172^ ^]^

These evidences suggest that SUMOylation of viral proteins opens a window for antiviral treatment, and downregulating viral SUMOylation could be potential antiviral treatment strategies. However, there are some potential problems that need attention. SUMOylation is involved in transcriptionally silencing of various latent viruses,^[^ ^173^ ^]^ thus the viral reactivation would be induced by SUMOylation inhibitors. The DNA damage response (DDR) pathway has been recognized to induce several antiviral cytokines, including IFN‐I, to restrict the replication of viruses.^[^ ^174^ ^]^ Interactions between viruses and DDR also have a significant impact on viral infection. It has been reported that multiple crucial proteins in DDR are SUMOylated.^[^ ^175^, ^176^ ^]^ Thus, SUMOylation inhibitors may interfere DDR pathway, causing accumulation of DNA damage, which would increase the risk of oncogenesis.^[^ ^177^ ^]^ Another factor that must be considered when proposing SUMOylation as an antiviral treatment is the role of SUMOylation in innate immunity. In some cases, SUMOylation is necessary for maintaining immune activation and homeostasis,^[^ ^102^, ^128^ ^]^ and the inhibition of SUMOylation therefore requires careful evaluation of its negative effects on antiviral innate immunity.

## 6 Conclusion and Perspectives

Viruses have evolved various strategies to establish a cellular microenvironment that is beneficial for their survival and reproduction by interacting with the SUMO machinery. Significant advances have been made in our understanding of the interplay between viruses and host SUMOylation pathways. However, we are only beginning to understand the impact of SUMOylation on viral replication and antiviral defense. Most viruses hijack the SUMOylation components of the host to modify their own proteins and promote viral replication. Some viral proteins are substrates for SUMOylation, which can negatively regulate their activities.^[^ ^48^, ^49^ ^]^ Viruses can also manipulate the cellular process of SUMOylation by disrupting the SUMO pathway. Detailed investigations into these mechanisms highlight the significance of SUMOylation processes in the viral replication cycle, and may reveal new targets for specific antiviral therapies.

SUMOylation also modulates the antiviral innate immune response of the host. SUMOylation is involved in regulating core signal molecules of the innate immune system, and it seems that SUMOylation can both enhance and suppress the antiviral innate immunity, depending on the substrate specificity and distinct site. Therefore, a thorough understanding of the role of SUMOylation in the innate immune response is crucial for preventing and treating virus‐associated diseases.

Viral infections are a major health concern worldwide, and can cause persistent infections and life‐threatening diseases. Targeting SUMOylation is a potential therapeutic approach for treating viral infection‐induced diseases. However, a key challenge is the translation of these findings into the development of new antiviral drugs that are suitable for human clinical applications. This challenge must be met to provide novel antiviral therapies in the future.

## Conflict of Interest

The authors declare no conflict of interest.

## Author Contributions

Y.F., X.L., and L.Z. contributed equally to this work. Y.F. conceived and drafted the manuscript. X.L. and L.Z. drew the figures and collected the information and summarized the table. Z.Z., F.W., J.H., and L.Z. discussed the concepts of the manuscript. H.Y., L.Z., and F.Z. provided valuable discussion and revised the manuscript.
